# Nanomechanics of Ultrathin Carbon Nanomembranes

**DOI:** 10.3390/nano13020267

**Published:** 2023-01-08

**Authors:** Marinos Dimitropoulos, George Trakakis, Nikolaus Meyerbröker, Raphael Gehra, Polina Angelova, Albert Schnieders, Christos Pavlou, Christos Kostaras, Costas Galiotis, Konstantinos Dassios

**Affiliations:** 1 Department of Chemical Engineering, University of Patras, GR-26500 Patras, Greece; 2 Institute of Chemical Engineering Sciences (ICE–HT), Foundation of Research and Technology Hellas, GR-26504 Patras, Greece; 3 CNM Technologies GmbH, Morgenbreede 1, 33615 Bielefeld, Germany

**Keywords:** carbon nanomembranes, mechanical properties, AFM

## Abstract

Ultrathin carbon nanomembranes (CNMs) are two-dimensional materials (2DM) of a few nm thickness with sub-nm intrinsic pores that mimic the biofiltration membranes found in nature. They enable highly selective, permeable, and energy-efficient water separation and can be produced at large scales on porous substrates with tuned properties. The present work reports the mechanical performance of such CNMs produced by p-nitrobiphenyl phosphonic acid (NBPS) or polyvinylbiphenyl (PVBP) and their composite membranes of microporous supporting substrates, which constitute indispensable information for ensuring their mechanical stability during operation. Measuring the nanomechanical properties of the ultrathin material was achieved by atomic force microscopy (AFM) on membranes both supported on flat substrates and suspended on patterned substrates (“composite membrane”). The AFM analysis showed that the CNMs presented Young’s modulus in the range of 2.5–8 GPa. The composite membranes’ responses were investigated by tensile testing in a micro-tensile stage as a function of substrate thickness and substrate pore density and diameter, which were found to affect the mechanical properties. Thermogravimetric analysis was used to investigate the thermal stability of composite membranes at high temperatures. The results revealed the structural integrity of CNMs, while critical parameters governing their mechanical response were identified and discussed.

## 1. Introduction

Water treatment processes are lately of high interest due to the emerging need to reduce water contamination and increase recycling. Among the different developed technologies [1,2], water separation technologies are widely applied throughout different sectors of modern industry and even in contemporary households [3,4] for the achievement of clean water and removal of unwanted substances. In pressure-driven membrane separation processes, the mechanical properties of the employed membranes are of particular importance because these materials carry the mechanical loads generated by the flow of liquids [5]. The membranes may be subjected to substantial physical tension at high working pressures, which can diminish or even destroy their performance, especially in the presence of inhomogeneities and defects [6]. When a membrane’s functional portion has poor mechanical qualities or its dimensions prevent it from operating at high pressures, one approach is to support it on porous reinforcing substrates of appropriate mechanical stability, hence forming so-called composite membranes [7]. The mechanical behavior of the *active layer* of the composite, as the supported CNM is termed, as well as of the whole composite membrane, is of increased interest [8,9,10].

CNMs are two-dimensional materials (2DMs) created by the cross-linking of self-assembled monolayers (SAMs) of aromatic precursor molecules using low-energy electron irradiation [11]. The thickness and porosity of CNMs can be adjusted by the choice of the precursor molecule type and processing parameters, which are responsible for the final membranes’ functionality [12,13]. CNMs created from the SAMs of terphenylthiol (TPT) precursor molecules (TPT-CNMs) have been shown to be highly permeable to water while exhibiting an extraordinarily high rejection of organic molecules, anions, and cations and are thus highlighted as ideal candidates for demanding water separation tasks [14,15]. Nonetheless, the handling and transferring of the nanometer-thin CNMs remain a tremendous scientific challenge. Recently, we developed a concept to synthesize mechanically stable supports with free-standing CNMs as active layers to directly create CNM-composite membranes [16]. This eliminates the involvement of critical steps such as the transferring of the nanometer thin CNM to the support, which is commonly found in other similar concepts (e.g., graphene-based membranes) [17,18]. The strategy is based on the track-etching technique [19] wherein an ion-beamed PET [poly(ethylene terephthalate)]-film of typically a few tens of µm thickness was used as a starting material. The ion bombardment leaves tracks, termed “*latent pores*”, at the locations where polymeric bonds in the PET are broken. Since the surface remains continuous, CNMs can still be created on these ion-beamed PET films. The tracks can be subsequently etched into open pores while the CNM, which is chemically inert, is not attacked. In fact, the membrane acts similar to an etch-stop and remains free-standing over the track-etched micron-sized pores in the PET support, while the production of such composite membranes is highly scalable [17,18].

Due to the unique characteristics of their anisotropic atomic structure and bonding, 2D materials, such as CNMs, display a wide range of unusual characteristics, while their exploitation relies greatly on the establishment of their mechanical attributes [20,21]. In contrast to macro-materials, the measurement of the mechanical properties of nanometer-thick membranes is particularly challenging. The nano-dimensions of such materials, as well as the difficulties associated with their handling, bring the available technology to its limits. There is currently a lack of certified experimental techniques for measuring the mechanical properties of 2D materials; such characterization usually relies on independent techniques which can offer the nanomanipulation capacities required for such tasks. One such technique is AFM, while experimental methodologies that indirectly provide mechanical performance indications, such as spectroscopic measurements, are also frequently employed [22,23,24]. 

In the present work, CNMs were suspended over patterned substrates, and their mechanical properties were evaluated quantitatively and directly at the nanoscale by AFM. Additionally, the mechanical properties of CNM-composite membranes supported on track-etched PET substrates were studied by tensile experiments using a micro-test tensile stage as a function of substrate thickness and pore density. The thermal stability of the composite membranes was studied at high temperatures by thermogravimetry. The current study gives insights into the acquired mechanical integrity of nanomembranes before and after their suspension over patterned substrates, thus highlighting the importance of a transfer-free procedure for the creation of nanocomposites for pressure-driven applications, as well as their thermal and mechanical stability as polymeric 2D active layers. The importance of the current work relies upon the use of AFM to collect quantitative and qualitative data on the mechanical properties of these nanomembranes, to offer a wide picture of their mechanical behavior more easily and with high accuracy. It could be shown that CNM-composite membranes have mechanical properties sufficient for pressure-driven water separation processes.

## 2. Materials and Methods

### 2.1. Synthesis of CNMs and CNM/PET Composites

In general, CNMs are prepared in a three-step process, as has been previously described in detail [11]. In brief, the process starts with a molecularly thin layer of aromatic precursor molecules deposited on a substrate by self-assembly, while dip-coating and spin-coating can also be used for the same scope. The layer is cross-linked under high vacuum conditions (pressure of ca. 10^−5^ mbar) with low-energy electrons of 50–500 eV at a dose of 50–100 mC/m^2^, which yields a highly cross-linked CNM that is mechanically, thermally, and chemically stable, and can be transferred from the initial substrate to virtually any other substrate, including porous ones, without collapsing. In this study, two types of membranes were prepared; namely, NBPS-CNMs were created by cross-linking self-assembled monolayers of p-nitrobiphenyl phosphonic acid (NBPS) (Figure S1) precursor molecules on aluminized PET-films (Al/PET) substrates [25], as well as membranes termed PVBP-CNM, created by the cross-linking of a few nanometer thick layers of polyvinylbiphenyl (PVBP) (Figure S2) on SiO_2_ substrates. These proprietary materials were supplied by CNM Technologies, Bielefeld, Germany.

CNM-composite membranes were prepared directly on top of PET substrates according to the following transfer-free recipe [16,25]. PET films of 23 and 36 µm thickness (SABEU GmbH & Co. KG, Northeim, Germany), which were bombarded by heavy ions for the creation of latent tracks, were coated with a few nanometers thickness of aromatic precursor molecules with a biphenyl backbone and subsequently crosslinked using the same energy and dose as in the previous case. Latent pores were opened from the tracks by etching with sodium hydroxide solution with a concentration of 5 M at a temperature of 80 °C. By varying the etching time within 10–15 min, the pore size could be adjusted within 0.5–25 µm. As the CNM active layer was present only on one side of the sheet and acted as an etch-stop, CNMs suspended freely over open pores of the PET substrate were obtained.

### 2.2. Transfer of CNMs on Substrates for AFM Measurements

**Figure 1:**
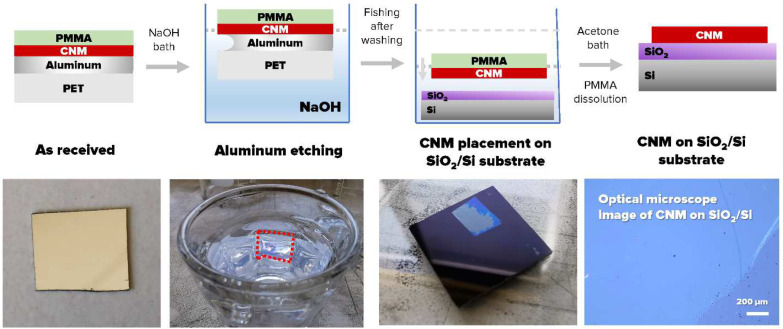
The transferring process of NBPS-CNM from Al/PET to Si.

As reliable nanomechanical measurements on nanometer-thick membranes in the AFM require flat-ideally, atomically flat-supporting surfaces [26], CNMs were transferred on solid SiO_2_/Si substrates as well as Si_3_N_4_ substrates perforated with 0.8 μm holes for testing the membranes in a suspended state. The transfer process was assisted by a sacrificial PMMA layer which stabilized the ultrathin CNM [25,27]; major characteristic steps in the process are depicted schematically in Figure 1 for the transfer of NBPS-CNMs from its original Al/PET growth substrate (for details, see [25]). Transfers of PVBP-CNMs from their SiO_2_/Si growth were performed by etching the SiO_2_/Si growth substrate, which was achieved in hydrofluoric acid.

### 2.3. AFM Characterization

All AFM characterizations were performed under ambient conditions in a Bruker Dimension Icon^®^ instrument residing in a noise-isolation chamber. Peak force-quantitative nano-mechanical (PF-QnM) mapping was used for the investigation of nanomechanical properties. The applied force was carefully selected so that the deformations occurring during the scan were purely elastic. PF-QnM offers the ability to acquire and analyze individual force curves from each tap on the material under investigation during the imaging process and extract the local mechanical properties [28]. To separate the contributions of different material properties, such as adhesion, Young‘s modulus, and deformation, it was necessary to measure the instantaneous force on the tip rather than a time-average of the force or dissipation over time, as is performed in the regular tapping mode. For quantitative results on the mechanical properties of the scanned surface, it was important to select a tip that could cause enough sample deformation while retaining high force sensitivity. Of paramount importance is also the correct calibration of deflection sensitivity and of a normal spring constant. In our experiments, silicon TESPA-V2 probes of radius *R* = 8 nm, the spring constant of *k* = 37 N/m, and resonance frequency of *f* = 320 kHz were used for topographic imaging. To obtain the optimal visualization of even the finer sample features, the forces exerted were kept to the lowest level possible. Nanomechanical data were also acquired and correlated to topography. To allow the acquisition of quantitative nanomechanical properties, the tip had to be calibrated for deflection sensitivity on a sapphire standard, while the Sader method was used for the force constant [29]. The same probes were used for the non-contact imaging of the suspended membranes.

### 2.4. Composite Tensile Tests on Track-Etched Substrates

The mechanical response of neat PET substrates and composite membranes in tension was recorded by corresponding testing on a Deben^TM^ micro-tensile stage, which is especially designed for measuring the mechanical properties of thin films [30]. The stage carries a load cell of 250 N, which allows high sensitivity on samples without high force demands. Samples were cut into strips of 30 mm × 2 mm and tested under a crosshead displacement rate of 1.5 mm/min. During the test, force-displacement pair values were continuously recorded, and stress–strain curves were derived. 

### 2.5. Thermogravimetric Analysis

The performance of polymeric materials, such as the substrate of the composite membranes, which constitutes the largest portion of the materials’ volume, was strongly influenced by the operational temperature and thermal transitions in the material. To investigate the thermal stability of the produced composite membranes at high temperatures, thermogravimetric analysis (TGA) experiments were performed in a TA Q50^®^ instrument (New Castle, DE, USA). Small pieces of the composite membranes were heated at 550 °C in a nitrogen atmosphere at a rate of 10 °C/min.

## 3. Results and Discussion

### 3.1. Mechanical Properties of CNMs

**Figure 2:**
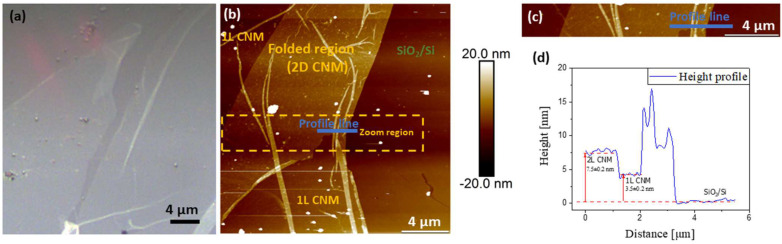
Representative AFM images of synthesized NBPS-CNMs. (**a**) Optical image, (**b**) AFM topography, (**c**) Magnified region, and (**d**) Height profile along the scan path defined in the magnified region. A folded bilayer CNM along the AFM scan path is defined by the height of the height line in blue color.

**Figure 3:**
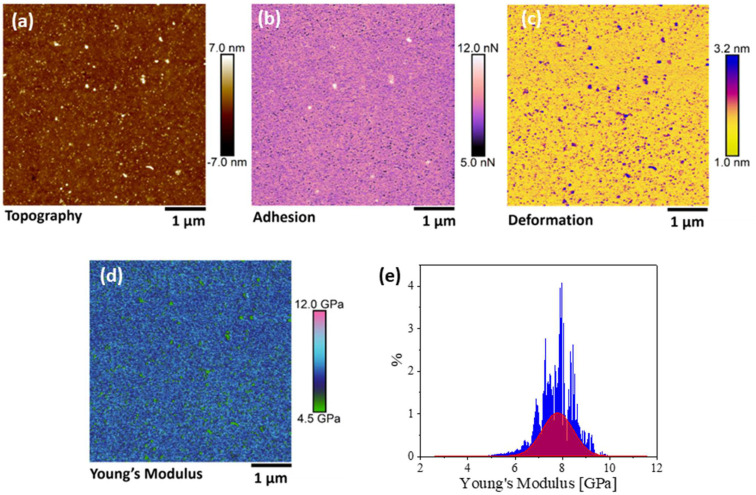
Typical nano-mechanical properties area of an area on a NBPS-CNM. The images display mappings of (**a**) Topography, (**b**) Adhesion, (**c**) Deformation, and (**d**) Young’s modulus. The white dots on the topography denote PMMA residues from the transfer of the membrane on the SiO_2_/Si substrate. (**e**) Histogram of the point-by-point analysis for the Young’s modulus mapping.

CNMs were transferred from their growth substrates to SiO_2_/Si and patterned Si_3_N_4_ substrates for topographic assessment and the measurement of mechanical properties at the nanoscale by AFM. Topography was obtained with both the contact and tapping mode AFM measurements, and the results showed good repeatability. Figure 2 shows the topography (Figure 2a–c) and height profile (Figure 2d) on an NBPS-CNM and along a scan path (blue arrows in Figure 2b,c) which contains a folded region of double height. The height of the scan path is presented in the blue color line in Figure 2d. The mappings of mechanical properties for a representative region are presented along with topography data in Figure 3. By the observation of the results therein, it is entailed that the topographic characteristics and mechanical properties of transferred CNMs exhibit very good homogeneity with an approximate roughness of 0.5 nm. Since the measurements took place within the elastic regime, energy dissipation for the CNM can be assumed negligible. PMMA residues hailing from the transfer process of the membrane on the SiO_2_/Si substrate were evident in all acquired channels and could be easily distinguished from the CNM due to their difference in nano-mechanical properties (e.g., white dots in Figure 3a,b). The tip-sample adhesion force was estimated at nearly 10 nN, while Young’s modulus ranged around a well-defined mean value of 8 GPa (Figure 3e).

**Figure 4:**
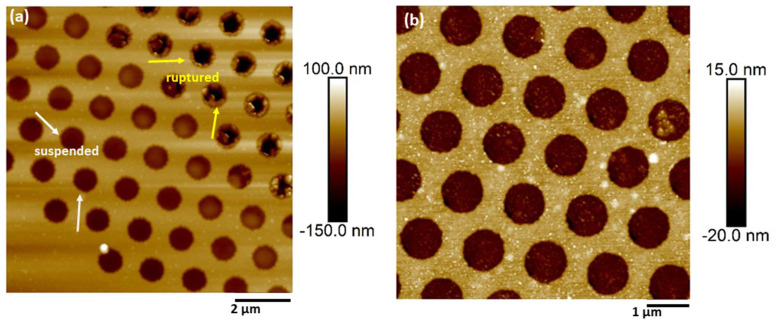
Freely suspended CNM membranes. The topography of suspended (**a**) Active layer of a CNM transferred from a composite membrane and (**b**) PVBP CNM.

**Figure 5:**
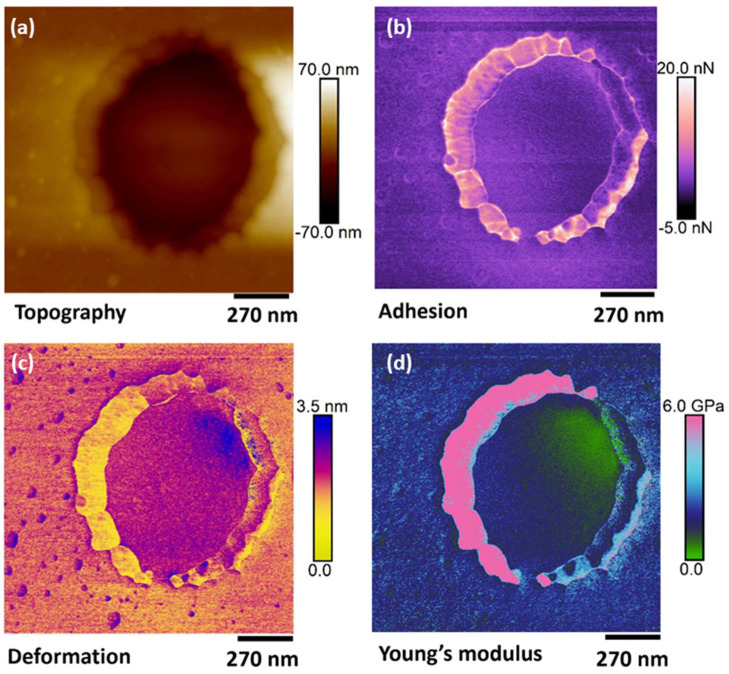
Representative mechanical properties of the active CNM-layer of a composite membrane in suspended state. Mappings of (**a**) topography, (**b**) adhesion, (**c**) deformation and (**d**) Young’s modulus.

As mentioned, free-standing CNMs were also nanomechanically characterized in the suspended state, except for pressure-driven separation applications, by transferring atop Si_3_N_4_ substrates regularly patterned with 0.8 μm diameter perforations. The suspended state allowed the acquisition of the neat mechanical properties of the membrane without the contribution of a substrate. The topography of the materials was acquired by a non-contact mode in order to minimize the interaction with the suspended materials [31]. It was observed that once the thin membranes were suspended on such perforated substrates, the surface forces attributed to van der Waals interactions between the substrate surface and supported membrane would cause the local adhesion of the membranes to the inner walls of the perforations of the supporting substrate, causing an apparent sagging [32]. As was observed from non-contact topographic mappings (Figure 4a), the transfer process of the thin membranes atop the voids of patterned substrates could also result in ruptured membranes. This “puncture of the drum” effect can be attributed to induced nonuniform coverage in combination with adhesive forces. The thickness of PVBP-CNMs was measured at 3 ± 0.5 nm, while the thickness of the active CNM layer of a CNM-composite membrane was measured at 12 ± 2 nm. Corresponding typical mappings of the mechanical properties for the active CNM layer of a composite membrane are shown in Figure 5, along with the respective topography. 

As evident from the mappings, the outer ring of the suspended material displays a different mechanical performance than the suspended region. This effect can be rationalized by the unequal tension applied by the rim to the suspended membrane, which differentiates it mechanically from the inside of the “drum”. Tip-sample adhesion appearing lower at the top-right regions inside the “drum” in Figure 5 indicates that the material may not adhere well to the substrate walls at that location. This discrepancy in adhesion gives rise to a difference in Young’s modulus for the suspended membrane, as observed in the profile distribution. This is also confirmed by the deformation channel, which shows higher values around the edge of the hole, signifying a region that is easier to distort under the same applied force. This uneven stress distribution and consequent downgrading of mechanical properties are detrimental to membrane applications as the particular sites are candidates for premature failure initiation. This finding signifies that transfer procedures play a critical role in the mechanical properties of the membrane system. Outside the drum region, the mechanical performance of the CNM demonstrates a high degree of homogeneity, as expected for an ideal interface, and the adhesion between the atomically flat Si_3_N_4_ substrate and the membrane. Young’s modulus at the region between the firmly clamped portion of the membrane (lower left) and the outside of the hole exhibit similar values. Young’ s modulus of the active CNM layer of the composite membrane ranged within 2.5–3 GPa. Similar behaviors were recorded for suspended PVBP CNMs, while those membranes of Young’s modulus were found to vary around a mean of 6 GPa. 

The very high modulus of elasticity that was found for NBPS (8 GPa) and PVBP (6 GPa) compared with common polymers can be attributed to their molecular structure. NBPS (Figure S1) and PVBP (Figure S2) are built upon a main polymeric chain of two benzene rings. The linearity of these chains and the presence of the rings offer high crystallinity and hence, high mechanical stability. On the other hand, the side functional groups present in the two polymers act competitively with the crystallinity, bestowing an increase in the amorphous density of the materials. With PVBP exhibiting larger chemical groups compared to NBPS, its crystalline-to-amorphous ratio is lower, resulting in an evident difference in Young’s modulus. It is worth noticing that Young’s modulus values are orders of magnitude higher than typical values of polymeric membranes used in forward osmosis applications or other water separation technologies (~dozens of MPa) [33,34,35,36,37,38]. In conclusion, it was found that the properties were strongly related to the geometrical characteristics of the substrates. This indicates that not only quantifying the mechanical properties of such materials is important, but also the geometry of the substrates should be taken into account for applications where the structural integrity of the material is crucial.

### 3.2. CNM-Composite Membrane Tensile Tests

**Figure 6:**
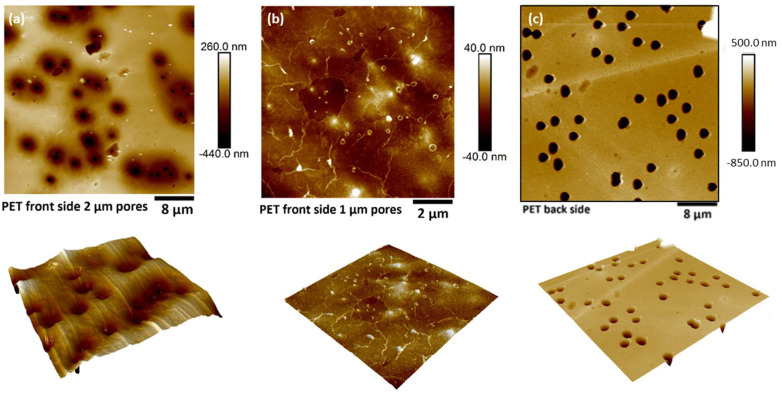
Representative topographies of CNM-composite membranes with PET supports of variable porosity. Topography of the front side showing suspended CNMs for pore sizes of (**a**) 2 μm and (**b**) 1 μm. (**c**) Topography of the back side (neat PET) used for pore diameter calculation. Additionally included are respective 3D AFM topographies.

**Figure 7:**
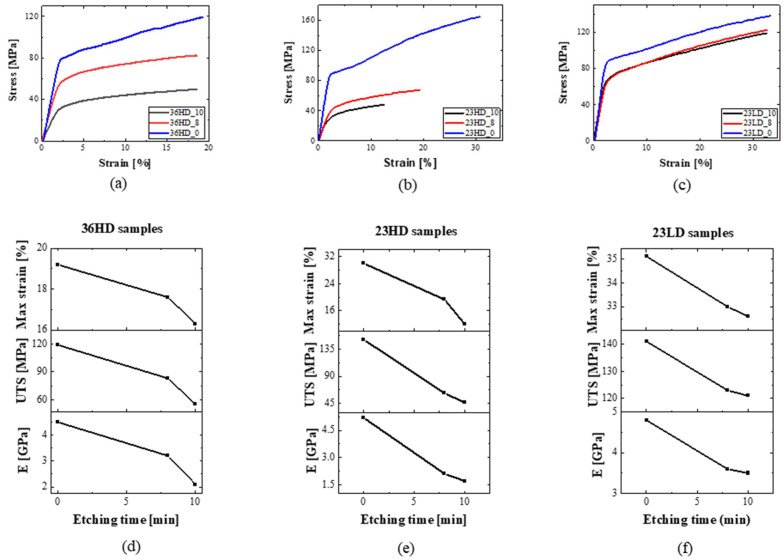
Characteristic stress–strain behavior of sample series (**a**) 36HD, (**b**) 23HD, and (**c**) 23LD. (**d**–**f**) Respective mechanical performance versus etching time.

As entailed from the AFM results of transferred CNMs, the transferring process on top of already-patterned substrates induce points of failure in the material system. In fact, this was the primary reason behind the need for the development of a transfer-free manufacturing process of CNM-composite membranes. Typical topographies of the front and back sides of the composites are shown in Figure 6. For pore diameters larger than 1 μm, the active CNM layer was found to display a downward curvature (sagging) inside the pore holes, which were slightly enlarged from their nominal value due to the local entrapment of etchant between the CNM and the PET substrate. The mechanical properties of CNM-composites, as functions of PET-support thickness, pore density, and pore size (quantified in terms of etching time), were investigated by tensile testing using the dedicated micro-tensile stage described in the experimental section. The pore diameter of each composite was assessed by non-contact mode AFM topography on the backside of the composite, where no CNM was present. Representative images of porous CNM composites are presented in Figure 6. Processing characteristics of the composites are shown together with sample coding in Table 1. The stress–strain curves of each family of samples, as well as the effect of the etching time on the mechanical performance, are presented in Figure 7. The engineering property values extracted from the curves are summarized in Table 2.

By observing Figure 7, it can be concluded that all three series of samples exhibited the characteristic mechanical behavior of polymers, i.e., demonstrated an elastic behavior at small strains followed by long plastic deformations at higher strains. The range of the observed engineering property values is of the same order of magnitude as of typical PET, i.e., ca. 100 MPa strength, 3.5 GPa Young’s modulus, and 20% failure strain. More specifically, the unetched specimens (series of samples with _0 suffix in the name) have, as expected, identical properties to pure PET with almost identical elastic regions and only slight differences in plastic behaviors. On the other hand, the tensile properties of the etched samples are found to downgrade with the substrate etching duration and, hence, also, pore size. Interestingly, the etched samples 23HD_10 and 23HD_8 present much inferior properties compared to 23LD_10 and 23LD_8, respectively. This occurs due to the increased etchant penetration into the higher porosity PET substrates, which, consequently, partakes in a higher volume of degradation of the composite.

**Figure 8:**
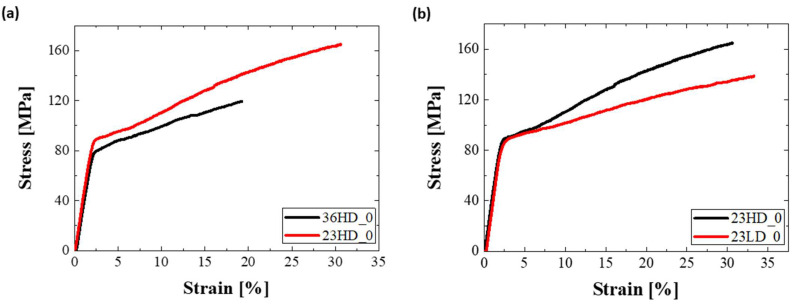
Tensile curves of unetched samples: effect of PET substrate (**a**) Thickness and (**b**) Pore density on mechanical behavior.

The effect of the sample thickness on mechanical performance is presented in Figure 8a, which makes it evident that decreasing PET substrate thickness (from 36 μm to 23 μm), has a positive impact on the tensile behavior: a finding which is of crucial significance for membrane scalability. This can be rationalized upon the total composite volume, wherein larger volumes (thicker samples) introduce more defects that are available to downgrade mechanical behavior. Furthermore, the mechanics of the composite system appear to be insensitive to pore density. By comparing the curves of etched samples 23HD and 23LD, it was found that a decrease in porosity of one order of magnitude, from 20 × 10^6^ pores/cm^2^ to 1.5 × 10^6^ pores/cm^2^, did not appear to affect the mechanical performance of the material albeit a slight effect only on the plastic region of the curves (Figure 8b). 

### 3.3. Thermogravimetry of CNMs/Substrates

**Figure 9:**
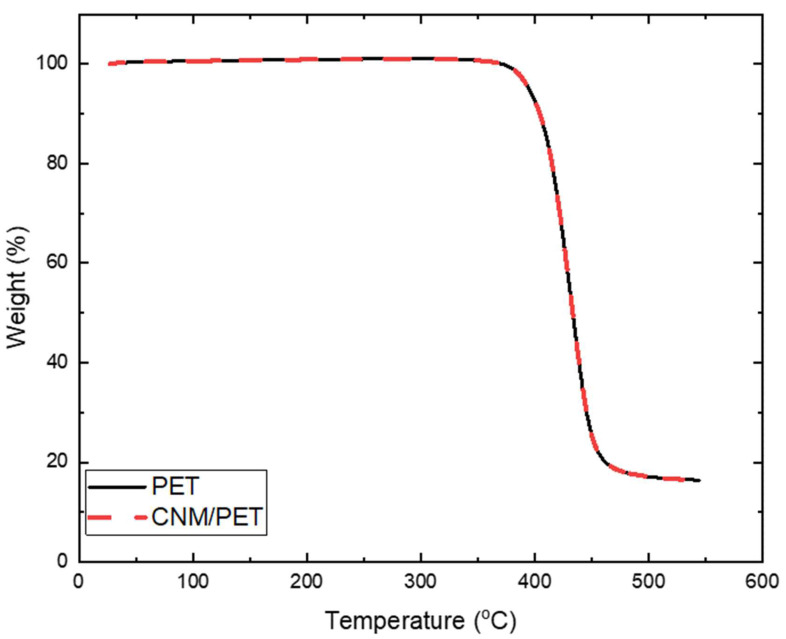
Thermogravimetric results of CNM/PET composites.

The typical TGA response of composite membranes and the PET reference sample is presented in Figure 9. As the CNM is a very small fraction of the composite, its contribution is minimal in the curve, which appears typical of neat PET materials [39]. The substrate is observed to be very stable until ~370 °C, where it starts to degrade. 

## 4. Conclusions

Two types of CNMs as model systems for pressure driven membrane water separation applications, each originating from different precursor molecules, NBPS and PVBP, were synthesized and tested in terms of their mechanical stability. NBPS-CNMs resemble, regarding their water separation performance, TPT-CNMs, which have been shown to be highly permeable for water while exhibiting an extraordinarily high rejection of organic molecules, anions, and cations; PVPB-CNMs are structurally similar to the active CNM-layer of our CNM-composite membranes [14,15]. AFM helped establish a clear picture of the nanomechanical behavior of CNMs on flat supported substrates and on suspended states atop perforated substrates, while composite membranes with micro-porous PET as supports of active CNM-layers were investigated at the macroscale. NBPS-CNMs exhibited well defined stiffness in terms of a Young’s modulus of 8 GPa while the property value for their PVBP counterparts was 6 GPa. The corresponding active CNM-layers of the composite membranes exhibited moduli in the region of 2.5–3 GPa. The findings indicate that the range of mechanical properties of the membranes can be adjustable by tuning the engineering parameters of the synthesis process. Moreover, it was found that the properties were strongly related to the geometrical characteristics of the substrates. The results indicate that free-standing CNMs over support pores of a few micrometers in diameter, as in CNM-composite membranes, are strong enough to withstand pressure-driven water separation processes. The mechanical properties of the whole CNM-composite membranes were of the same order of magnitude as those of neat PET films (the material of the support layer). This finding was expected by the consideration of the small volume fraction of CNMs in the composites. Etching time, which determined the diameter of pores in the PET-support of the composites, was established as a key factor in the mechanical degradation of the substrates. A significant role of mechanical properties was also played by substrate thickness, with thinner substrates demonstrating better mechanical performance due to a lower defect density. These results are an indication, that the mechanical properties of the support, which is governed by the thickness, pore density and pore diameters, might actually be the limiting factor in pressure-driven water separation and not the mechanical stability of the CNM-active layer. Pore density did not appear to have an important effect on the unetched samples, while high porosity downgraded the samples in accelerated degrees. The thermal stability of the composites was found to be almost identical to PET, denoting that the major part was played by the substrate of choice due to the negligible volume of the active layer. The current work proves that the QnM technique can be a very helpful tool for quantifying both quantitative and qualitative mechanical details of nanomembranes.

## Figures and Tables

**Table 1:** Processing parameters of the three types of porous PET substrates etched for duration of 0 min (not etched), 8 min, and 10 min, together with sample coding: (i) thickness of 36 µm with high pore density (HD), (ii) thickness of 23 µm with high pore density (HD), and (iii) thickness of 23 µm with low pore density (LD).

Sample	Etching Time (min)	Thickness (μm)	Pore Density (cm^−2^)
36HD_10	10	36	20 × 10^6^
36HD_8	8	36	20 × 10^6^
36HD_0	0	36	20 × 10^6^
23HD_10	10	23	20 × 10^6^
23HD_8	8	23	20 × 10^6^
23HD_0	0	23	20 × 10^6^
23LD_10	10	23	1.5 × 10^6^
23LD_8	8	23	1.5 × 10^6^
23LD_0	0	23	1.5 × 10^6^

**Table 2:** Engineering property values extracted from the stress–strain curves.

Sample	Young’s Modulus (GPa)	Ultimate Strength (MPa)	Strain (%)
36HD_10	2.1 ± 0.3	55 ± 8	16.3 ± 2.0
36HD_8	3.2 ± 0.6	83 ± 9	17.6 ± 2.5
36HD_0	4.5 ± 0.4	119 ± 11	19.2 ± 2.7
23HD_10	1.7 ± 0.3	46 ± 4	12.0 ± 2.0
23HD_8	2.1 ± 0.3	62 ± 12	19.3 ± 0.5
23HD_0	5.2 ± 0.7	159 ± 14	30.1 ± 1.5
23LD_10	3.5 ± 0.3	121 ± 8	32.6 ± 2.5
23LD_8	3.6 ± 0.7	123 ± 3	33.0 ± 1.7
23LD_0	4.8 ± 0.2	141 ± 12	35.1 ± 2.8

## Data Availability

The data that support the findings of this study are available upon reasonable request from the authors.

## Supplementary Materials

The following supporting information can be downloaded at: https://www.mdpi.com/article/10.3390/nano13020267/s1, Figure S1: Molecular structure of NBPS. Figure S2: Molecular structure of PVBP.
