# Supplementary material for: Nanomechanics of Ultrathin Carbon Nanomembranes
Source: Nanomaterials (Basel). 2023 Jan 8;13(2):267. doi: 10.3390/nano13020267 (PMC9863011; doi:10.3390/nano13020267)
Supplement: Supplementary file 1 [file nanomaterials-13-00267-s001.zip › nanomaterials-2091053-supplementary.pdf]

Supporting Information

Nanomechanics of Ultrathin Carbon Nanomembranes

Marinos Dimitropoulos [1,2], George Trakakis [1,2], Nikolaus Meyerbröker [3], Raphael Gehra [3], Polina Angelova [3], Albert Schnieders [3], Christos Pavlou [1,2], Christos Kostaras [1,2], Costas Galiotis [1,2] and Konstantinos Dassios [1,2,*]

* Correspondence: kdassios@upatras.gr

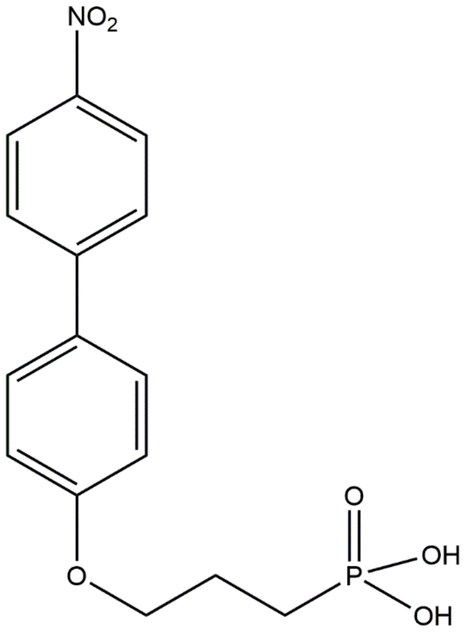

Figure S1: Molecular structure of NBPS.

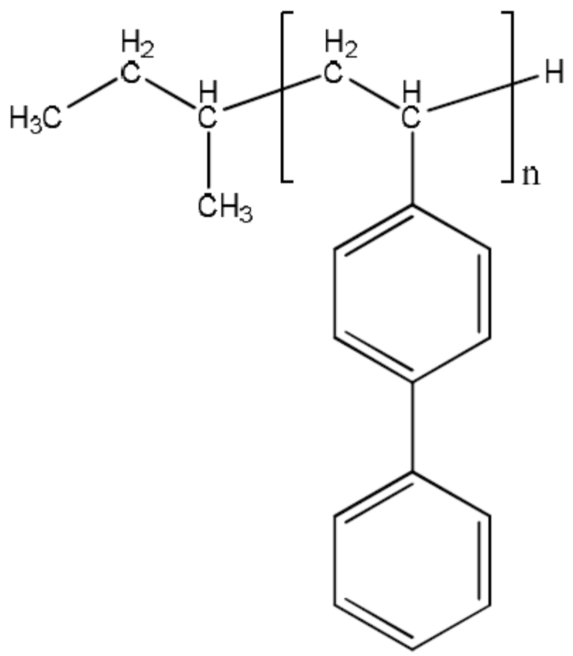

Figure S2: Molecular structure of PVBP.
